# Evolution of language: Lessons from the genome

**DOI:** 10.3758/s13423-016-1112-8

**Published:** 2016-07-18

**Authors:** Simon E. Fisher

**Affiliations:** 1 Language and Genetics Department, Max Planck Institute for Psycholinguistics, Wundtlaan 1, 6525 XD Nijmegen, The Netherlands; 2 Donders Institute for Brain, Cognition and Behaviour, Radboud University, 6525 EN Nijmegen, The Netherlands

**Keywords:** Genetics and genomics, Speech and language, Evolution, Ancient DNA, Model systems

## Abstract

The post-genomic era is an exciting time for researchers interested in the biology of speech and language. Substantive advances in molecular methodologies have opened up entire vistas of investigation that were not previously possible, or in some cases even imagined. Speculations concerning the origins of human cognitive traits are being transformed into empirically addressable questions, generating specific hypotheses that can be explicitly tested using data collected from both the natural world and experimental settings. In this article, I discuss a number of promising lines of research in this area. For example, the field has begun to identify genes implicated in speech and language skills, including not just disorders but also the normal range of abilities. Such genes provide powerful entry points for gaining insights into neural bases and evolutionary origins, using sophisticated experimental tools from molecular neuroscience and developmental neurobiology. At the same time, sequencing of ancient hominin genomes is giving us an unprecedented view of the molecular genetic changes that have occurred during the evolution of our species. Synthesis of data from these complementary sources offers an opportunity to robustly evaluate alternative accounts of language evolution. Of course, this endeavour remains challenging on many fronts, as I also highlight in the article. Nonetheless, such an integrated approach holds great potential for untangling the complexities of the capacities that make us human.

Our speech and language capacities enable us to acquire vocabularies of many thousands of words, assemble them into a myriad of structured meaningful expressions, and convey thoughts to others by mapping meaning to sound, and back again. In the twenty-first century, we are witnessing dramatic advances in deciphering the genetic architecture underlying these fascinating aspects of the human condition. By directly borrowing state-of-the-art gene mapping approaches from studies of typical biomedical traits, and applying them to scientific studies of language for the first time, it has become feasible to start tracing out relevant genetic networks (Graham & Fisher, 2015). The language sciences are thereby witnessing a paradigm shift, moving far beyond prior speculative models in which genes have been invoked as abstract entities with mysterious powers, an issue that I have discussed in detail elsewhere (Fisher, 2006; Fisher & Vernes, 2015). Gene discovery strategies take advantage of the modern human population as a kind of natural experiment (e.g. Narasimhan et al., 2016) for uncovering potential connections between genotype (the genetic constitution of an individual at a particular locus or set of loci) and phenotype (the appearance of that individual in terms of a particular characteristic, be it physical, biochemical, physiological, behavioural, etc.). Applying this framework to the language sciences entails searching for statistically significant correlations between variations observed at the genomic level and variability in speech- and language-related skills, with the aim of establishing causal relationships.

Due to rapid progress in molecular methods, we now have a particularly comprehensive view of the ways that genes and genomes vary from one person to the next (see The 1000 Genomes Project Consortium, 2015). Genetic variations range from mutations that are extremely rare (perhaps even unique to one individual or family) all the way through to common variants that are found in populations at high frequency. Rare mutations can have severe effects on gene products, for instance by preventing an important protein from being made or interfering with its function, and could thereby be sufficient to cause a major disorder affecting one or more tissues of the body. Common variants (also known as polymorphisms) tend to have much more subtle effects on gene function, for example by leading to a slight change in the quantity or activity of the protein that a gene codes for. Indeed, many genetic polymorphisms are completely benign, and those that do have biological effects typically show probabilistic relationships with phenotypic outcomes, for example by partially altering the risk of a particular disease or accounting for a tiny difference in a quantitatively defined trait (height, blood pressure, body-mass index, and so on). Thus, a person’s phenotype can be considered the consequence of combinatorial effects of all the rare and common gene variants that their genome carries, together with their interactions with the environment, as well as stochastic factors. In contrast to the current wealth of information on genotypic variability in human populations, we know considerably less about the nature of phenotypic variability in speech and language skills. (Perhaps this is in part due to the emphasis that linguists have traditionally placed on universals.) In principle, searching for genotype-phenotype correlations is an approach that can be applied across the entire spectrum of variability observed in modern humans, but, thus far, most emphasis has been placed on studies of pathology (Newbury & Monaco, 2010).

## Opening molecular windows

A major focus of the field has concerned neurodevelopmental disorders in which children suffer from disproportionate impairments in mastering aspects of speech and language, against a background of relatively preserved cognitive function, and despite adequate exposure to language input in their environment (Bishop, 2001). It has long been known that such unexplained disorders of speech and language development tend to cluster in families, and are highly heritable (that is, a substantial proportion of risk is due to genetic factors). Studies of DNA samples from families with rare forms of these disorders have allowed geneticists to go further and pinpoint the specific genetic disruptions that are causal (Graham, Deriziotis, & Fisher, 2015). The most often cited example is the identification of a *FOXP2* mutation causing speech apraxia, along with expressive and receptive language impairments, in multiple generations of a large British Caucasian family, referred to in the literature as the KE family (Lai, Fisher, Hurst, Vargha-Khadem, & Monaco 2001). However, this is certainly not the only mutation to have been clearly implicated in this type of disorder. Additional families and unrelated cases have been found carrying different causative mutations of *FOXP2*, and rare disruptions in other genes, such as *ERC1* and *BCL11A*, have been reported in children with a similar profile of severe speech and language problems (see Graham & Fisher, 2015 for a detailed overview of the latest findings). In addition, studies of common genetic variation have begun to identify polymorphisms that may make more subtle contributions to language pathology; for example, putative risk variants of the *CNTNAP2* gene have been associated with reduced performance on language tasks in children with specific language impairment (Vernes et al., 2008).

In studying genetic underpinnings of speech and language capacities, the successful isolation of a gene that contributes to the relevant phenotype is not the endgame. To the contrary, it is a starting point—the value of implicating a specific gene in a trait lies in the novel avenues of investigation that can be followed as a consequence. The identification of a gene like *FOXP2* opens up unique molecular windows into both the neural bases and the evolutionary origins of speech and language (Fisher & Scharff, 2009; Scharff & Petri, 2011). I will consider each of these lines of research in turn below.

## Insights into neural pathways

Regarding neural bases, an array of sophisticated experimental approaches, ranging from analyses of human neurons grown in a dish (Vernes et al., 2007), through to studies of circuits and behaviour in genetically manipulated animal models (French & Fisher, 2014; Wohlgemuth, Adam, & Scharff, 2014), can reveal fundamental roles of the gene of interest in brain development and function. These methods may appear relatively new for scientists studying language, but they are established mainstays of molecular neuroscience, developmental neurobiology and other related fields. Indeed, some language scientists may be surprised to learn that the general principles governing how genetic programs help build a complex nervous system are already well worked out (reviewed by Fisher & Vernes, 2015). The products of genes (RNA molecules and proteins) interact with each other to mediate the proliferation of cells that will become neurons, their differentiation into particular types of neurons, and migration of these neurons during development to their final locations in the brain (Tan & Shi, 2013). Moreover, connectivity patterns in the central nervous system emerge from a tight interplay of genetic and environmental factors—gene products underlie mechanisms by which projections emerge from the neuronal cell body to become dendrites and axons, as well as the growth and guidance of axons towards their target neurons (Kolodkin & Tessier-Lavigne, 2011). Even the strengthening or weakening of the individual connections between neurons (synapses), the basis of learning and memory, depends critically on activities of certain sets of genes (Holtmaat & Svoboda, 2009).

Having pinpointed a gene involved in a language-related disorder, purely based on genomic data from families and cases, researchers can then delve deeply into the functional correlates to uncover how the implicated gene impacts on neuronal proliferation, differentiation, connectivity, plasticity, and so on, drawing from the growing set of elegant experimental tools and systems that molecular neuroscience has to offer (Fisher & Vernes, 2015). In addition, the mutations that yield speech and language impairments can be directly introduced into cells grown in the laboratory, or into animal models, to help understand how the crucial mechanisms and pathways go awry in disorder. For example, the *FOXP2* mutation that causes a severe speech and language disorder in the KE family is a change to a single letter of DNA, leading to alteration of the amino-acid sequence of the encoded protein. Genetic engineering makes it possible to create and study human neurons that carry this same change, or even insert the identical mutation into another species, such as a mouse, an issue I return to later.

Before moving on, three important take-home messages from molecular neurobiology are worth emphasising. First, clearly a gene does not itself specify a particular behaviour output, nor does it even specify a particular neural circuit. The pathways by which molecular factors impact on neural circuitry and cognitive functions are by their very nature indirect and must occur via intermediate effects on the types of neurobiological processes discussed above (proliferation, differentiation, connectivity, plasticity, etc.). The necessarily complex mappings from gene to behaviour mean that discussions that centre on an abstract “gene for language” are unconstructive; more nuanced accounts built on biological principles give an opportunity for real progress (Fisher, 2006). Second, typically a gene does not have a single restricted function, but instead contributes to more than one process, is active in a range of distinct cell-types, and/or plays roles at multiple developmental time points or in different environmental contexts. This widespread property of gene action is usually referred to by the technical term of *pleiotropy*. The same gene can thus have multiple roles within the brain, as well as contributing to development and function of non-neural tissues. Given that the human genome comprises only ~20,000 protein-coding genes, it is perhaps unsurprising that each gene is “re-used” in a number of different contexts in the brain and body, with the precise functions of the encoded protein depending on the other proteins that are active in the tissue. This leads to the third take-home message, which is that genes and proteins do not act in isolation but interact with each other in networks and complexes. Indeed, the combinatorial nature of gene activity is a highly valuable feature for researchers interested in deciphering the biology underlying a trait of interest. When *FOXP2* was first identified, it quickly became clear that this gene encoded a type of protein that, working together with other related proteins, regulates how certain sets of genes (downstream targets) are switched on and off. In other words, *FOXP2* represents a hub in a genetic network. The tools of molecular biology have since enabled the identification of additional elements of this network, including interactors, like *FOXP1* and *TBR1*, and downstream targets, such as *CNTNAP2*, that are also implicated in neurodevelopmental disorders and language-related traits (Deriziotis et al., 2014; Sollis et al., 2016; Vernes et al., 2008). In general, as our understanding of gene networks becomes more and more sophisticated, this holds great promise for helping to successfully bridge the gap between the genome and the brain.

## Towards empirical studies of evolution

Identification of genes implicated in speech and language disorder also provides valuable entry points for empirical studies of evolutionary origins of human traits, rooted in biological data (Enard, 2011; Fisher & Marcus, 2006). By taking the DNA sequence of a known language-related gene (and the amino-acid sequence of its protein product) and comparing it to corresponding sequences found in different species across the animal kingdom, it is possible to reconstruct the likely evolutionary history of the gene, determining a time window when it first emerged, the nature of alterations along different lineages, and whether it has shown a distinctive profile of change in our most recent ancestors. (To be clear, here I focus on what we can learn from comparative analyses of extant species, and address the promise of ancient hominin data in a later section of this article.) It is also feasible, although more difficult, to characterize where and when the gene product is active in different structures of the developing and adult human brain (based on analyses of post-mortem tissue), and compare this neural expression pattern to that seen in other species. If potentially significant evolutionary differences are detected in a gene of interest, this can yield hypotheses about functional impact that can be empirically evaluated using model systems (Enard 2014).

Again, studies of *FOXP2* give a nice illustration of the concept. Comparative analyses revealed that, far from being unique to humans, this gene has a deep evolutionary history and is present in similar form in distantly related vertebrate species (Scharff & Petri, 2011). Conservation has been found not only at the DNA/protein sequence level but also in assessments of neural expression patterns (e.g. Bonkowsky & Chien, 2005; Haesler et al., 2004; Kato et al., 2014; Lai, Gerrelli, Monaco, Fisher, & Copp, 2003; Teramitsu, Kudo, London, Geschwind, & White, 2004), with findings also supported by experiments assessing gene function in several different species (French & Fisher, 2014; Wohlgemuth, Adam, & Scharff, 2014). Such data suggest that the gene has ancient roles in the development and function of certain brain circuits involving the cortex, basal ganglia and cerebellum, with relevance for sensorimotor integration and motor-skill learning (e.g. French et al., 2012; Groszer et al., 2008; Haesler et al., 2007; Murugan, Harward, Scharff, & Mooney, 2013). Against this background of high evolutionary conservation, the human version of *FOXP2* carries two amino-acid coding changes that occurred after splitting from the common ancestor with the chimpanzee, leading to a specific hypothesis that one or both of the evolutionary changes might have been important for the emergence of speech and language on our lineage (Enard et al., 2002). Crucially, researchers have gone on to test this hypothesis using the same model systems and experimental approaches that are used for investigating mutations that cause disorder. For example, when the key evolutionary amino-acid changes were inserted into genetically modified mice, the mice showed higher levels of plasticity of synapses in cortico-basal ganglia circuits (reviewed by Enard, 2011). By contrast, when mice were genetically modified to carry a disruptive *FOXP2* mutation that is known to cause a speech and language disorder (the mutation from the KE family) such mice showed *lower* levels of synaptic plasticity in cortico-basal ganglia circuits, consistent with a loss of function (see Groszer et al., 2008). Thus, it seems that the experiments with mice carrying the evolutionary changes are capturing something about the biological significance of those changes, rather than simply reflecting disturbance of existing pathways. Investigations of evolutionary history have also been used to assess recent positive selection of broader networks regulated by *FOXP2* (Ayub et al., 2013) and to evaluate other candidate genes implicated in language-related phenotypes, such as *KIAA0319*, *ROBO1*, *ROBO2*, and *CNTNAP2* (Mozzi et al., 2016).

## Learning from our genomes

The accumulated data from molecular studies support the view that genetic underpinnings of speech and language skills are highly multifactorial, indicating that no single locus is sufficient by itself to account for such traits (Graham & Fisher, 2015). In particular, the genes that have been most clearly implicated in relevant developmental disorders can explain only a small subset of affected families and cases. The majority of discoveries have thus far depended on laborious detective work using a standard genetics toolkit, along with some serendipity in targeting unusual families and cases with monogenic forms of disorder. However, the advent of next generation DNA sequencing means that we can now sequence the whole genome (or at least a substantial proportion of it) in any human individual for less than 1000 euros, in a matter of days, and requiring only a sample of saliva. This development holds considerable promise for increasing our knowledge of the genetic aetiology of rare forms of speech and language disorder. Already, sequencing of the entire coding parts of the genome (the exome) is beginning to make its mark on the field (e.g. Villanueva et al., 2015). At the same time, even cheaper methods enable rapid high-throughput screening of hundreds of thousands of common genetic variations for less than 100 euros per person, allowing for large-scale genome-wide studies of common forms of language-related disorders (Gialluisi et al., 2014) and even investigations of normal variation in the general population (Luciano et al., 2013; St Pourcain et al., 2014). It will also be interesting to study people at the other extreme of the phenotypic spectrum, such as those rare individuals who have exceptional abilities to master many languages. Of course, the availability of inexpensive accessible techniques for capturing genomic variation leads to its own new challenges, primarily in distilling biologically meaningful signals from vast datasets, but creative solutions are in place, or on the horizon (see Graham & Fisher, 2015 for further discussion). These efforts will yield further candidate genes and associated networks for targeted analyses of neural function and evolutionary history.

## Insights from ancient hominin DNA

Advances in genomics are not only transforming gene-mapping studies of modern day humans. In one of the most astonishing technological achievements of molecular biology, it is now possible to read off sequences of nuclear DNA from ancient organisms that are extinct, giving an unprecedented glimpse into the genomes of the past (Pääbo, 2014). Molecular anthropologists have successfully sequenced entire genomes of ancient hominins using archaeological samples estimated to be tens of thousands of years old (Fu et al., 2014; Meyer et al., 2012; Prüfer et al., 2014). Sequenced genomes are available not only for ancestors on our own lineage, such as a ~45,000-year-old modern human (Fu et al., 2014), but also for >50,000-year-old bones from independent hominin branches, including Neandertals (Prüfer et al., 2014) and Denisovans (Meyer et al., 2012). Note that, although the samples themselves are tens of thousands of years old, the time-depth they provide for comparative evolutionary analyses is much greater, because the common ancestor of modern humans and Neandertal/Denisovan hominins existed several hundred thousand years ago. Most recently, nuclear DNA sequences have been recovered from two hominin samples that are estimated to be >430,000 years old, although DNA of this age is much too degraded to ever yield a complete genome sequence (Meyer et al., 2016).

Ancient hominin DNA sequences, together with matching data from extant primates, provide invaluable additional datapoints for evaluating the evolutionary significance of changes in language-related genes. As before, *FOXP2* provides an interesting case in point. Examination of the two amino-acid coding changes that distinguish the human sequence from that of chimpanzees reveals that they are not unique to humans (Krause et al. 2007) but also present in Neandertal and Denisovan samples. Some have taken this as one of the supporting points in favour of the view that our Neandertal cousins also possessed some form of spoken language (Dediu & Levinson, 2013), although it is important to stress that the status of a single gene is not enough to resolve whether or not an ancient hominin could speak. Further in-depth comparisons of modern human and Neandertal versions of *FOXP2*, examining the parts of the genetic locus that do not code for protein, identified human-specific changes that might potentially affect the way that the gene is regulated (Maricic et al., 2013). Although the functional data to support this hypothesis are tentative at present (Maricic et al., 2013), this work represents another example where ideas about evolutionary impact do not remain speculation, but are open to empirical testing.

## Weighing up alternatives

Importantly, the availability of virtually complete genome sequences for modern and ancestral humans, Neandertals, Denisovans and great apes, allows molecular anthropologists to generate genome-wide catalogues of evolutionary changes that occurred on the different lineages during distinct evolutionary periods (Pääbo, 2014). These comparative molecular catalogues are of enormous value for both constraining and enhancing accounts of the origins of human traits, including (but not limited to) our linguistic capacities. For example, certain hypotheses concerning the origins of language posit the existence of just a single causative DNA mutation, occurring on the human lineage after splitting from the other hominin branches, and most probably within the last 100,000 years (Chomsky, 2011; Crow, 1997; Klein & Edgar, 2002). Other accounts consider the evolutionary emergence of proficient spoken language as a multistage process involving multiple phenotypic components and multiple genomic changes (Fitch, 2012), with some hypotheses placing importance on gene–culture interactions (Fisher & Ridley, 2013; Laland, Odling-Smee, & Myles, 2010). Now that we have access to comprehensive descriptions of genomic changes along different branches of the hominin tree, it will become feasible to empirically evaluate different evolutionary accounts, to assess which are more compatible with the available molecular data.

For example, based on analyses of ancient genomes, it has been estimated that 96 amino-acid changes, in 87 protein-coding genes, have become fixed on the human lineage (that is, they are now shared by all humans in every population) after splitting from our common ancestor with Neandertals (Prüfer et al., 2014). Such comparisons also identified ~3,000 fixed changes, outside of protein-coding regions, with potential to impact on the regulation of gene expression, arising during this same period. Future research programmes can use bioinformatics, functional analyses in neuronal cell models, comparison to data from language disorders, and other screening methods, to systematically assess the likely functional impact of the various changes of interest, and their relevance to neural phenotypes. If it is assumed that Neandertals did not have language (a controversial issue as noted above) and that the unusual linguistic capacity of modern humans is the result of just a single relatively recent mutation of large effect (also subject to considerable debate), then it will very likely be contained within our existing catalogue of fixed human–Neandertal differences. Thus, although I am personally sceptical of such a single-mutation account, if this model is correct then the putative responsible mutation is entirely open to discovery via experimental means. To be clear, I do not doubt that sorting through the many plausible genomic changes is a difficult challenge, certainly at present when the available functional assays are still laborious and low-throughput. However, the key point is that this research programme (and other similar endeavours) is in principle a perfectly tractable one, well within the grasp of modern science. By paying attention to the relevant genomes themselves, we move away from unconstrained speculation about abstract genetic factors, to empirical evaluation of alternative perspectives on the origins of language.

It is important to recognize that language origins present us with an inverse problem; we will never precisely reconstruct the evolutionary history of our species. However, given the new sources of empirical data that can be brought to bear on the emergence of human traits, we are in a much stronger position to distinguish between the merits of different accounts of language evolution, and to generate novel hypotheses that are amenable to experimental testing.
